# Magnetic turbulence in a table-top laser-plasma relevant to astrophysical scenarios

**DOI:** 10.1038/ncomms15970

**Published:** 2017-06-30

**Authors:** Gourab Chatterjee, Kevin M. Schoeffler, Prashant Kumar Singh, Amitava Adak, Amit D. Lad, Sudip Sengupta, Predhiman Kaw, Luis O. Silva, Amita Das, G. Ravindra Kumar

**Affiliations:** 1 Department of Nuclear and Atomic Physics, Tata Institute of Fundamental Research, Colaba, Mumbai 400005, India; 2 Group for Lasers and Plasmas, Instituto de Plasmas e Fusão Nuclear, Instituto Superior Técnico, Universidade de Lisboa, 1049-001 Lisboa, Portugal; 3 Institute for Plasma Research, Gandhinagar 382428, India

## Abstract

Turbulent magnetic fields abound in nature, pervading astrophysical, solar, terrestrial and laboratory plasmas. Understanding the ubiquity of magnetic turbulence and its role in the universe is an outstanding scientific challenge. Here, we report on the transition of magnetic turbulence from an initially electron-driven regime to one dominated by ion-magnetization in a laboratory plasma produced by an intense, table-top laser. Our observations at the magnetized ion scale of the saturated turbulent spectrum bear a striking resemblance with spacecraft measurements of the solar wind magnetic-field spectrum, including the emergence of a spectral kink. Despite originating from diverse energy injection sources (namely, electrons in the laboratory experiment and ion free-energy sources in the solar wind), the turbulent spectra exhibit remarkable parallels. This demonstrates the independence of turbulent spectral properties from the driving source of the turbulence and highlights the potential of small-scale, table-top laboratory experiments for investigating turbulence in astrophysical environments.

## Abstract (web summary)

Understanding the role of magnetic turbulence in the atmosphere is difficult as direct access is limited, but latest laser technology can enable such studies in the lab. Here the authors probe the evolution of such turbulence in laser-generated plasma with its implications to astrophysical environments.

From planetary magnetospheres and stars to primordial structures, turbulent magnetic fields are ubiquitous^1^,^2^. They play a dramatic role in energetic events such as triggering violent energy release through magnetic reconnection and coronal heating^2^ and are pivotal in controlling confinement in magnetic fusion devices^3^. Yet, in spite of orders-of-magnitude variations in the characteristic plasma parameters, the universality of turbulence dictates commonalities between these diverse manifestations. Herein lies the beauty of turbulence. One may therefore wonder about the parallels between various astrophysical turbulent scenarios and an experimentally-accessible, turbulent laser-generated plasma. Where does magnetic turbulence originate and how does it evolve? Table-top laboratory experiments can play a crucial role in finding answers to these compelling, fundamental questions^4^,^5^,^6^,^7^.

The first demonstration of magnetic turbulence in an intense-laser-generated plasma was reported in our previous work^8^. These measurements were confined to less than 8 picoseconds (ps) of turbulent magnetic field evolution, and could be understood within an electron magnetohydrodynamic (EMHD) framework.

Here, we illustrate the transition of the turbulent magnetic fields in an intense laser plasma from an initial, purely electron-mediated turbulent regime to a subsequent regime, where ions and electrons are both involved. In this latter regime, a gradual emergence of a spectral kink in the turbulent magnetic-field spectra is observed. This separates two distinct power-law spectral scalings, which become progressively more prominent at ion gyroperiod timescales (several tens of ps in our laboratory experiment). Spectral breaks demarcating different turbulent regimes have also been observed in solar flare loops^9^, the solar photosphere^10^, as well as the solar wind^11^,^12^,^13^,^14^,^15^ (arguably the best laboratory for studying astrophysical turbulence). However, the energy injection in our laboratory experiment is through the electron species, whereas that in the solar wind is through ion free-energy sources. Our table-top laboratory measurements thus closely reproduce the astrophysical observations, despite originating from very different mechanisms of energy injection.

## Results

### Experimental set-up and methodology

**Figure 1:**
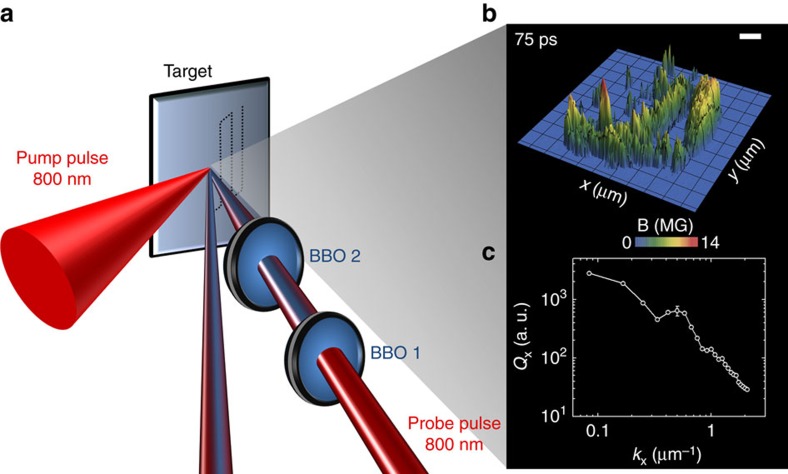
Experimental set-up and representative magnetic-field polarigram and turbulent energy spectrum. (**a**) Schematic of the experimental set-up. The intense driving laser (pump) pulse at a central wavelength of 800 nm and with a pulsewidth of 30 femtoseconds (fs) and a peak irradiance of 3 × 10^18^ W cm^–2^ was focused on a millimetre-thick aluminium-coated BK7-glass target, creating the turbulent megagauss magnetic fields in the plasma. The temporal and spatial evolution of the magnetic fields were then monitored using a time-delayed third-harmonic probe pulse, generated using a pair of beta-barium-borate (BBO) crystals. (**b**) Typical transverse (*x*−*y*, along the target surface) profile of the magnetic field *B* at the point of interaction, measured 75 ps after the incidence of the driving laser pulse, exhibiting the highly filamentary structure of the megagauss (MG) magnetic fields. The scale bar corresponds to 10 μm. (**c**) The corresponding energy spectrum of the turbulent magnetic field at 75 ps, clearly depicting the spectral kink. Here, 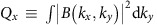 and *k*_*x*_ ≡ 2*π*/*x* and so on and the results are symmetric in *x* and *y*. Each spectrum is the average of several laser shots, with the error bar denoting the s.d.

Figure 1 shows a schematic of the experimental pump-probe polarimetric set-up, along with a representative magnetic field polarigram and its turbulent magnetic-energy spectrum. (For details on the polarimetric measurement of the magnetic fields, mapped with micron-scale optical resolution and sub-picosecond-scale temporal precision, see Methods section) The study of the transition from a purely EMHD regime to a regime where ions play a significant role was enabled by employing an ultraviolet third-harmonic probe pulse (at a wavelength of 266 nm), leading to a reduced background noise at long timescales (until 75 ps), in comparison to a visible second-harmonic probe pulse (at 400 nm) employed in our earlier experiments (confined only to 8 ps)^8^.

### Temporal evolution of turbulent energy spectra

**Figure 2:**
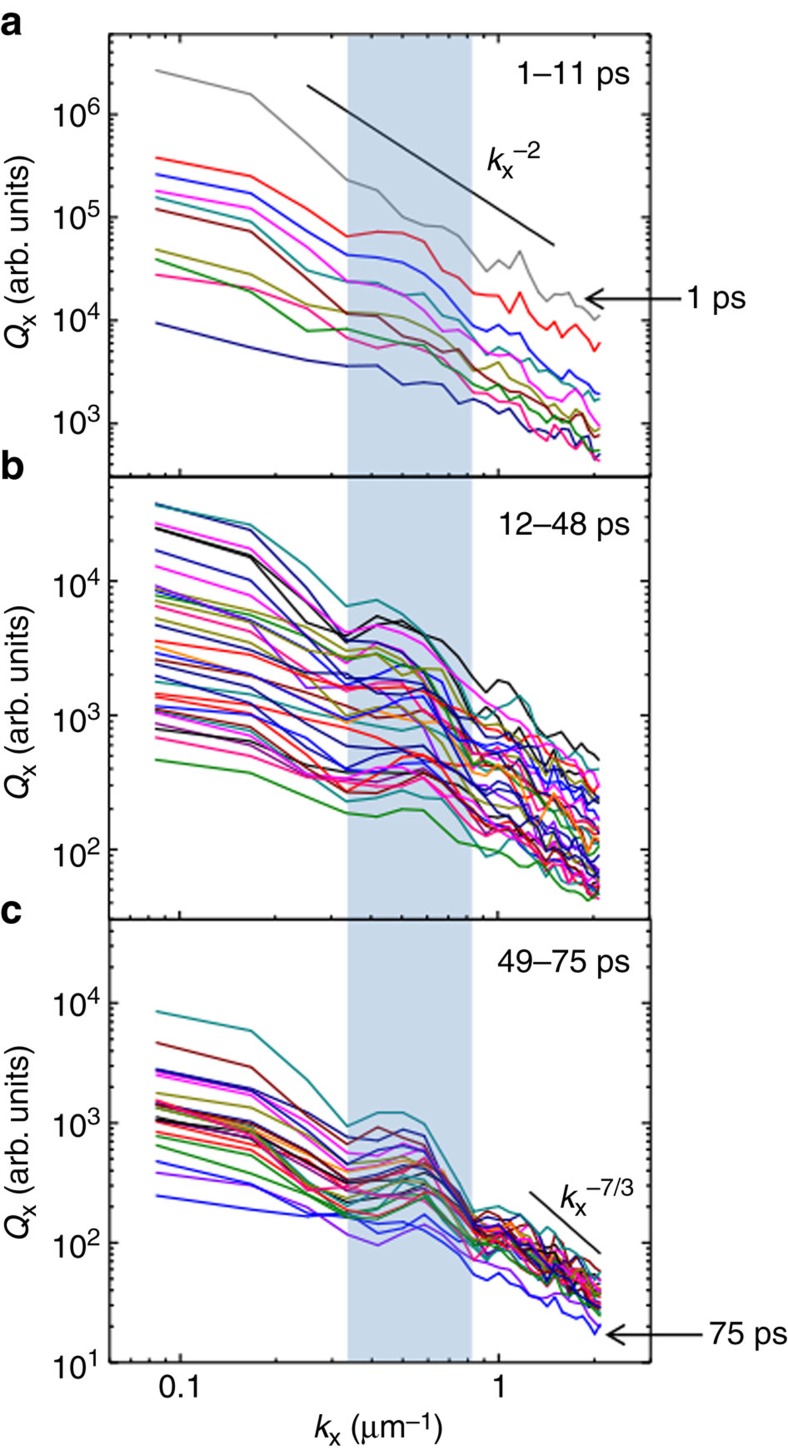
Temporal evolution of the magnetic-energy spectra. Each magnetic-energy spectrum denotes a specific temporal delay between the pump and probe laser pulses, ranging from 1 to 75 ps with a temporal resolution of 1 ps, and averaged over several laser shots. As in Fig. 1, 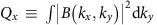 and *k*_*x*_ ≡ 2*π*/*x* and so on and the results are symmetric in *x* and *y*. (**a**) Despite the highly filamented, turbulent magnetic field profiles, the energy spectra show a distinct power-law behaviour (∝*k*^−*α*^) at the initial timescales with a spectral index *α*≈2. (**b**,**c**) At later timescales, there is a transition in the spectral index from *α*<2 (for small *k*_*x*_) to *α*>2 (for large *k*_*x*_), separated by a gradually emerging spectral kink (accentuated by the blue vertical bar).

Figure 2 shows the temporal evolution of the turbulent energy spectra of the megagauss magnetic fields. The data have been acquired from thousands of laser shots in experiments repeated and re-examined over the course of two years for varying laser as well as target conditions. At initial timescales of a few picoseconds, the magnetic-energy spectra depict a distinct power-law behaviour (as shown in Fig. 2a). This is consistent with a spectral index of *α*≈2 (where the wave-number *k* scales as *k*^−*α*^). Subsequently, a spectral kink emerges and becomes progressively more pronounced at timescales of a few tens of picoseconds, distinguishing two spectral regimes of turbulence (as shown in Fig. 2b,c). The rather gradual formation of the spectral kink inhibits an exact specification of its time of appearance with picosecond precision. However, a reasonable estimate concurs with timescales corresponding to the inverse of the ion cyclotron frequency, 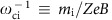
≳12 ps for an average magnetic field *B*∼20 MG and an effective degree of ionization *Z*≲12 for an aluminium target (consistent with our experimental observations). Here, *m*_i_ and *e* are the ion mass and electronic charge, respectively. (See Methods section for further details on the estimation of the ion temperature *T*_i_ and *Z*, consistent with time-resolved shadowgraphy measurements.) Besides, an estimation of the ion gyroradius *ρ*_i_ ≡ *v*_th,i_/*ω*_ci_, where *v*_th,i_ is the thermal velocity of the ion, dictates the spectral location of the kink at *k*∼2 μm^−1^, according to the relation 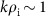. This is not significantly different from the experimentally observed value of *k*∼0.5 μm^−1^, given the choice of the average magnitudes of the magnetic field and the ion thermal velocity in the theoretical estimate (both spatially and temporally varying, dynamic parameters in the experiment).

## Discussion

The underlying mechanism leading to the observed turbulent spectra may be understood as follows. The incidence of an intense femtosecond-duration laser pulse (with peak irradiances exceeding 10^18^ W cm^–2^) on a solid target generates mega-ampere relativistic hot electron currents. The forward propagation of these currents is prevented by the self-generated magnetic fields, in accordance with the Alfven limit, first proposed in the astrophysical context^16^,^17^,^18^. The ambient plasma, created by the ionization of the solid by the intense laser pulse, produces current-neutralizing return flows of cold electrons. These return currents facilitate the forward propagation of the energetic relativistic electrons by reducing the net current to a value below the Alfven limit. The interaction between the counter-propagating high-current electron beams, however, is susceptible to a host of electromagnetic instabilities^19^, ultimately leading to the turbulent fragmentation and filamentation of the electron beams.

The observations during the first few picoseconds of the experiment may be understood within an EMHD framework^8^,^20^. In this regime, electron-beam-induced instabilities are expected to generate magnetic fields (for instance, the Weibel instability^19^,^21^). However, the Weibel instability alone would inject power at the typical scale of the electron skin-depth, where the growth rate maximizes. If this were indeed the dominant instability in the experiment, the spectral power would have initially been only in the neighbourhood of the electron skin-depth (typically sub-microns for our experimental parameters). The experiments, however, show spectral power at long scale-lengths as early as *t*∼1 ps. To investigate whether nonlinear effects could explain the observations at early timescales at long scale-lengths, particle-in-cell (PIC) simulations (using the OSIRIS code^22^) were carried out (Supplementary Fig. 1 and Supplementary Discussion). The simulations showed the development of the Weibel instability, although its nonlinear inverse cascade was found to be too slow to account for the experimentally observed spectral power at long scale-lengths. Therefore, alternative mechanisms were explored to investigate whether power could be directly injected at the long scale-lengths. Additional PIC simulations were performed (also using the OSIRIS code^22^) with the choice of a finite transverse extent for the incident laser beam. These simulations clearly showed significant spectral power in the magnetic field at the scale-length of the transverse beam extent from the very beginning (Supplementary Fig. 2 and Supplementary Discussion). We thus conclude that in the early EMHD regime, the shear-flow-driven instabilities arising due to transverse velocity gradients at the edges of the finite-sized electron beams^16^,^17^ tend to be dominantly excited linearly. These driving processes typically occur over a timescale of 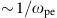 (typically sub-femtosecond for our experimental conditions), where *ω*_pe_ is the electron plasma frequency.

In the EMHD regime, which lasts until *t*≲12 ps in our experiments, the nonlinear cascade of magnetized electron instabilities establishes a spectral power with a scaling index of *α*≈2, which is the theoretically predicted estimate^23^. This is consistent with the experimentally observed slope of the power spectra in Fig. 2a at these initial timescales. The spectral power is observed to be maximum at *t*=1 ps, when the instability already saturates and the energy injection stops. The turbulence, thereafter, takes a decaying character.

At later timescales (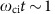, where *t*≳12 ps), the appearance of the spectral kink at scale-lengths defined by 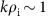 (as shown in Fig. 2b,c) provides indication towards the initiation and growing prevalence of ions over the initial, purely electron-mediated turbulent regime. At the long spatial scale-lengths 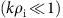, the magnetization of both the ions and the electrons leads to a magnetohydrodynamic (MHD) plasma turbulence, marked by a spectral index *α*<2. In contrast, the small spatial scale-lengths 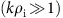 are marked by a spectral index of *α*>2, typical of the nonlinear cascade in the Kinetic Alfven Wave (KAW) regime^24^. The transition in the value of the spectral index *α* consequently induces the conspicuous spectral break in the observed magnetic turbulence. There are numerous examples of similar observations made in spacecraft measurements of solar wind turbulence, where a spectral kink has been reported separating the MHD and KAW turbulent regimes^11^,^13^,^14^,^15^. This reiterates that the spectral properties of MHD and KAW turbulence are universal, dictated entirely by the nonlinear processes involved. Furthermore, they are independent of the details of energy injection, namely electron-mediated instabilities in our laser-plasma experiments and ion-dominated instabilities in the astrophysical context. (See Methods section for a discussion on the magnetic Reynolds number for our experimental parameters.)

In summary, our experiment follows the progression of magnetic turbulence in an intense-laser-generated plasma from an electron-mediated turbulent regime to a regime where ion-magnetization plays a dominant role. This ion-dominated regime is in striking resemblance with the magnetic turbulence observed in spacecraft measurements of the solar wind. This suggests that turbulence is invariant across scales varying over several orders of magnitude, and independent of channels of excitation and dissipation. In light of the ubiquity of turbulence, we are thus encouraged to envisage a controlled laboratory environment that may simulate and tailor dynamic, universal turbulent mechanisms, ranging from tokamaks to turbulent cascades in solar magnetic reconnection geometries.

## Methods

### The experimental set-up and methodology

The experiment was performed with a 20 terawatt, chirped-pulse-amplified titanium-doped sapphire-based intense laser. The main interaction pulse of duration 30 fs and peaked around a central wavelength of 800 nm was focused with an off-axis parabolic mirror to a focal spot size of 12 μm × 15 μm on a millimetre-thick aluminium-coated BK7-glass slab to produce peak irradiances of 3 × 10^18^ W cm^–2^. A time-delayed probe laser pulse, derived from the main interaction pulse, was converted into its third harmonic at a central wavelength of 266 nm using a pair of beta-barium-borate (BBO) crystals. A third-harmonic probe can penetrate to near-solid densities of ∼10^22^ cm^–3^ in the plasma at near-normal incidence, nearly an order of magnitude higher than the critical density of the main interaction pulse (Fig. 1a). The incident probe was focused loosely to a spot-size diameter of ∼75 μm on the target, while the reflected probe was channelled through ultraviolet-sensitive high-extinction-ratio Glan-Taylor polarizers into a set of ultraviolet-sensitive charge-coupled-devices coupled with narrow-bandpass interference filters allowing radiation at 266 nm. The optical resolution of the imaging system, calibrated with a standard USAF-1951 target, was measured as 3.1 μm, which, along with the probe focal-spot diameter on the target plane (∼75 μm), specified the spectral range for our observations. Further details on the methodology of mapping the spatial and temporal evolution of the megagauss magnetic fields by pump-probe Cotton-Mouton polarimetry can be found in our previous work^25^.

The temporal delay of the probe with respect to the main interaction pulse was initially varied until 8 ps with a 200-fs resolution, and the experiment was then repeated until a temporal delay of 75 ps with a 1-ps resolution. In addition, the experiment was also repeated for bulk aluminium and bulk copper targets employing a second-harmonic probe, peaked at a central wavelength of 400 nm, under otherwise identical experimental conditions. Qualitatively similar turbulent features could be observed, although the data were more prominent for the third-harmonic probe rather than the second-harmonic one. This is because the strong self-generated second harmonic of the laser from the plasma amplifies the background noise in the detection system, while using a second-harmonic probe. This effect is even more prominent at longer timescales, when the reflected probe is weak. In contrast, the third-harmonic background noise was found to be non-perturbative and negligible for all timescales, while using a third-harmonic probe.

The magnetic field polarigrams obtained by the measurement of the Stokes’ parameters of the incident and the reflected probe^25^ were fast-Fourier-transformed with a rectangular windowing function. Other standard windowing functions (such as Hann, Hamming, Gaussian and triangular) were found to produce qualitatively similar results with the spectral kink even more pronounced.

### Determination of the effective degree of ionization

One-dimensional MULTI-fs^26^ hydro simulations were used to estimate the effective degree of ionization *Z* of the target. The effect of a 1-ns prepulse at an intensity of 3 × 10^13^ W cm^–2^ (consistent with an experimentally measured, laser-intensity contrast of 10^−5^) was simulated on a bulk (510-μm thick) aluminium target. The main interaction pulse with a pulsewidth of 30 fs and at an irradiance of 3 × 10^18^ W cm^–2^ was made to interact with the preplasma generated by the prepulse. A temperature of ∼750 eV was obtained in the underdense plasma, rapidly decreasing with increasing density while approaching the bulk of the initially cold solid target. According to FLYCHK^27^ simulations, temperatures of ≲750 eV are consistent with a degree of ionization of *Z*≲12. The expansion speed *c*_*s*_ of the plasma calculated from the above parameters was found to be consistent with time-resolved shadowgraphy measurements of the expanding plasma under identical experimental conditions. The simplistic model described here is aimed at obtaining only an approximate order-of-magnitude estimate for the ion cyclotron frequency *ω*_ci_ and the ion gyroradius *ρ*_i_.

### Model for spectral scaling

The experimental observation of an initially single spectral index (*α*≈2), followed by the evolution of a spectral break separating two distinct turbulent regimes with disparate spectral indices (*α*<2 and *α*>2), may be understood on the basis of the dynamic roles played by the electrons and the ions at various stages of evolution of the magnetic turbulence.

At initial timescales 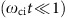, the laser energy is fed in to the electrons, which are responsible for driving the magnetic turbulence. This turbulence is driven both at the electron skin-depth scale *d*_*e*_ due to the Weibel instability, as well as at the long spatial scale-lengths corresponding to the transverse extent of the hot electron beam due to velocity shear at the edges. The latter has been corroborated by PIC simulations (Supplementary Fig. 2 and Supplementary Discussion). These long scale-length magnetic fields act as the ambient background for the whistler-mediated EMHD cascade, which produces the broad turbulent spectra of magnetic excitation. This leads to the *α*≈2 scaling^23^, close to our experimental observations (as shown in Fig. 2a). It should be noted that the ion-beam-mediated Weibel instabilities and shocks play no role in our experiment. This is because the estimated growth time for these instabilities, given our experimental parameters, is more than the duration of the experiment.

At later timescales 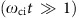, the ion response becomes significant. Furthermore, if the spatial scale-length under consideration includes the ion gyroradius *ρ*_i_, the physics in the two regions separated by this scale-length is significantly different. For a scale-length longer than the ion gyroradius 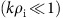, Alfven-like MHD perturbations may be expected in the magnetic-field spectrum. The typical power spectrum for strong turbulence in this regime^1^ scales as *α*≈5/3, consistent with our experimental observations, as shown in Fig. 2c. The slope in this regime is shallower than that of the *α*≈2 scaling, which was observed throughout during the electron-mediated regime in our experiments (as shown in Fig. 2a).

For a scale-length shorter than the ion gyroradius 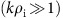, the equations describing Alfvenic perturbations are significantly altered because warm plasma effects have to be retained. Here, the kinetic effects in the ion dynamics become crucial and the regime is aptly termed KAW regime. The turbulent spectra in this regime is believed to have a scaling of *α*≈7/3 or *α*≈8/3 (see, for instance, refs 24, 28 and references therein). Both these scalings are steeper than *α*≈2, consistent with our experimental observations (as shown in Fig. 2c). It may be noted that the resistive scale falls beyond the maximum value of *k* depicted in Fig. 2. The spectral curve for 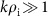 follows a power-law and can be easily distinguished from an exponential resistive decay 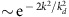, where *k*_*d*_ denotes the resistive wave-number and should take a value in the range 0.5–2 μm^−1^, if the resistive scale is to lie within our spectral range of observation.

A conservative estimate for the magnetic Reynolds number for our experimental parameters is 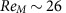, whereas that of the solar wind^29^ is typically 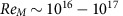. The inertial range of turbulence, therefore, shows similar properties in systems with magnetic Reynolds number greater than unity. In systems with 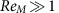, the number of decades in the wave-number over which the inertial range can operate is very large, which obviously imposes rather impractical demands on laboratory experiments.

### Data availability

All relevant data are available from the authors on request.

## Additional information

**How to cite this article:** Chatterjee, G. *et al*. Magnetic turbulence in a table-top laser-plasma relevant to astrophysical scenarios. *Nat. Commun.* **8,** 15970 doi: 10.1038/ncomms15970 (2017).

**Publisher’s note**: Springer Nature remains neutral with regard to jurisdictional claims in published maps and institutional affiliations.

## Supplementary Material

Supplementary Information

Supplementary Movie 1

Supplementary Movie 2

Peer Review File
