# Supplementary material for: Magnetic turbulence in a table-top laser-plasma relevant to astrophysical scenarios
Source: Nat Commun. 2017 Jun 30;8:15970. doi: 10.1038/ncomms15970 (PMC5497062; doi:10.1038/ncomms15970)
Supplement: Supplementary Information [file ncomms15970-s1.pdf]

Type of file: pdf
Size of file: 0 KB
Title of file for HTML: Supplementary Information
Description: Supplementary Figures and Supplementary Discussion.

Type of file: MOV
Size of file: 0 KB
Title of file for HTML: Supplementary Movie 1
Description: **Particle-in-cell (PIC) simulation output for the temporal evolution of magnetic fields.** Out-of-plane and in-plane magnetic fields are represented by colour and arrows respectively.

Type of file: MOV
Size of file: 0 KB
Title of file for HTML: Supplementary Movie 2
Description: **Particle-in-cell (PIC) simulation output for the temporal evolution of the magnetic-energy spectra.** The red and black represent out-of-plane and in-plane magnetic fields respectively, and blue represents the magnetic field perpendicular to the other two.

Type of file: pdf
Size of file: 0 KB
Title of file for HTML: Peer Review File
Description:

I. SUPPLEMENTARY FIGURES

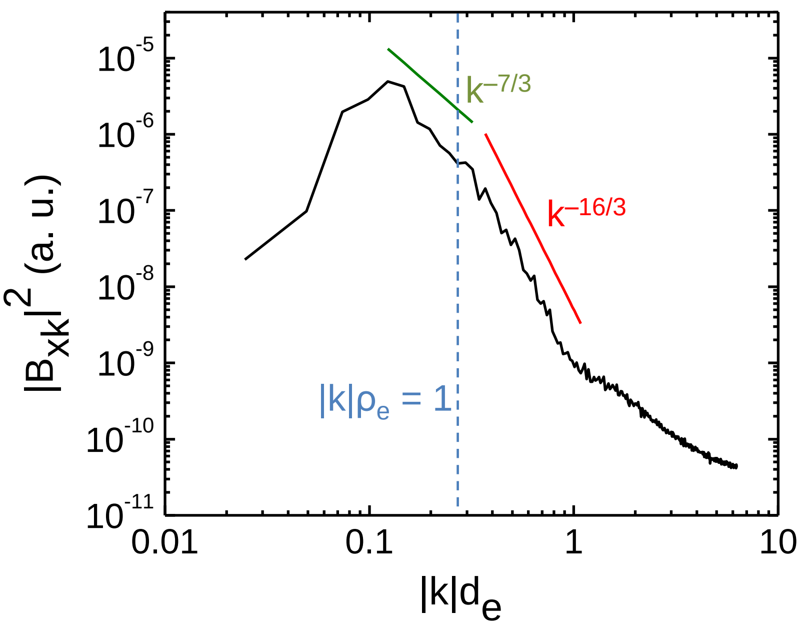

Supplementary Figure 1. Particle-in-cell (PIC) simulation output using OSIRIS at time $t = 14280\omega_{pe}^{-1} \approx 8$ ps. The magnetic-energy spectrum exhibits power-law behavior and consists of a peak at $|k|d_e = 0.16$, corresponding to filaments of size $\sim 40d_e$, where d_e is the electron skin-depth. A spectral break is also observed at $|k|\rho_e = 1$, where ρ_e is the electron gyroradius.

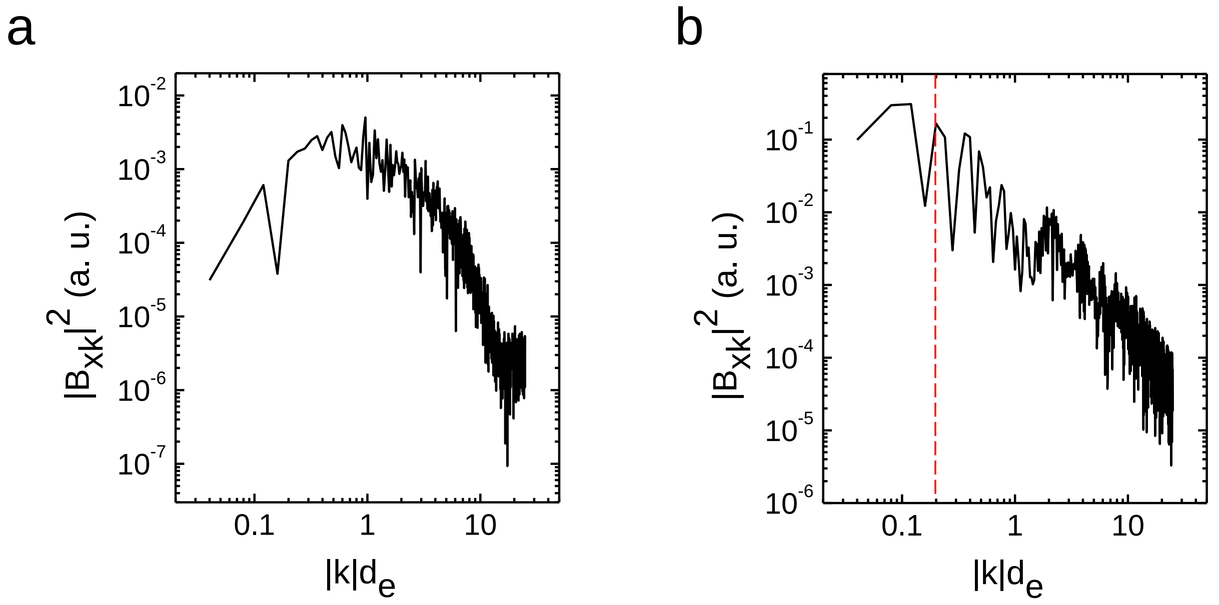

Supplementary Figure 2. Effect of a beam of finite transverse extent on the particle-in-cell (PIC) simulation output at $t = 36\omega_{pe}^{-1} \approx 20$ fs. a, The beam fills the entire simulation box. **b,** The beam is of finite transverse extent, represented by the red-dotted line.

II. SUPPLEMENTARY DISCUSSION

A full-scale, *ab initio* simulation of the filamentary instabilities leading to the excitation of turbulent magnetic fields and the onset of ion involvement is too demanding. It would necessitate three-dimensional particle-in-cell (PIC) simulations capable of spatially resolving the micro-scale ($\sim c/\omega_{pe}$) instabilities, yet extending up to the macro-scales ($\sim 360c/\omega_{pe}$) defined by the spatial extent of turbulent excitation (sampled by the experimental probe). Furthermore, the simulations must be continued for several picoseconds ($\sim 10^4\ \omega_{pe}^{-1}$) of plasma evolution – a computational feat too formidable even for current state-of-the-art computing facilities. Consequently, we developed a reduced, simplified numerical model, while retaining the most essential physics of magnetic field generation and evolution due to the Weibel instability. Two-dimensional particle-in-cell (PIC) simulations were performed using the OSIRIS framework. OSIRIS is a state-of-the-art, massively parallel, fully relativistic electromagnetic multidimensional PIC code. In OSIRIS, a set of computational particles is moved under the action of their self-consistent electromagnetic field and any externally applied field. This is achieved by first depositing the current density of the computational particles on a spatial grid. Maxwell's equations are then solved on the same grid, and the force acting on each particle is computed, interpolating the field values on the position of the point particle. The particles are then advanced under this force, and the PIC loop is closed by depositing again the current density of the particles in their new positions and with their updated momenta.

In our system, a spatially uniform beam of relativistic electrons with proper velocity $0.5c$ (c being the speed of light) and density $0.1n_0$ (where n_0 is the total electron density) was introduced in a background with current-neutralizing return currents and charge-neutralizing ions. The mass ratio m_i/m_e between the ions and the electrons was reduced to 100 in order to reach ion time-scales more quickly. The thermal velocity was defined as $v_{\rm th} = \sqrt{T/m}$, where T and m are the respective temperature and mass of the species. Consequently, the thermal velocities for the electrons and ions were considered to be $0.1c$ and $0.01c$ respectively.

The simulation was performed in a $256d_e \times 256d_e$ box, where $d_e \equiv c/\omega_{pe}$ is the electron skin-depth. Periodic boundary conditions were imposed. A resolution of 16 grid cells per d_e was used with 64 particles per grid cell for the return current and ions, and 32 particles per grid cell for the stream electrons. The spectrum of each of the components of the magnetic

field was considered as a function of k. The Fourier transform of the magnetic field was multiplied by the complex conjugate to obtain the energy spectra, and then averaged over all directions of $\mathbf{k}$ to obtain the spectra as a function of the absolute value of k. The simulations were continued until $t = 14280\omega_{pe}^{-1}$ (approximately 8 ps).

The complete evolution of the magnetic fields, along with the coalescence of the filaments, is provided in Supplementary Movie 1, which shows the out-of-plane magnetic fields (color) and the in-plane magnetic fields (arrows).

The evolution of the magnetic field power spectra has been shown in Supplementary Movie 2. The red represents the out-of-plane field, the black represents the field along the flow direction, and the blue represents the field perpendicular to the other two.

Considering the real mass-ratio in the simulations would introduce a larger range of scales between the ion and electron physics. As long as this range is sufficiently long, the dynamics of the plasma will not change. In our case, the spectra between the ion Larmor-radius scale ($kd_e = 0.025$) (where the Larmor radius is defined using the typical velocity perpendicular to the magnetic field B, and a typical magnitude of B) and the electron Larmor radius ($kd_e = 0.25$) is large enough to see a power-law behavior.

The simulations clearly show that the initial spectra maximize around the electron skin-depth. There is evidence of inverse spectral cascade towards longer scale-lengths. However, despite simulations running for several thousands of plasma period, there is no evidence of the spectral power reaching out to the ion gyroradius ρ_i (or even at the longest observable scale-length of the system in the nonlinear regime). This is in stark contrast with the experimental observations, where the spectral power seems to be maximum at the longest observable scale-length from the very beginning (as early as $t \sim 1$ ps). It should be noted that the longest observable scale-length corresponds to the focal spot-size of the probe laser beam, which is five times that of the pump laser pulse. This led us to consider the possibility of other driving mechanisms, which can inject power at longer scale-lengths. The possibility of electron velocity shear at the beam edge (the typical size of which is commensurate with the focal spot of the pump laser pulse) playing a role in this regard was recognized. To investigate this, another set of simulations was performed, taking into account the finite size of the electron beam, using OSIRIS. The results are shown in Supplementary Fig. 2, which shows a comparison of similar runs for a beam filling the entire simulation box of size $25d_e$ as well as a beam with a finite transverse extent $5d_e$, which is 1/5 of the simulation box chosen.

In the former scenario, the spectral power maximizes at the electron skin-depth ($|k|d_e = 1$). On the contrary, in the latter scenario, the maximum power occurs at the scale-length of the transverse beam size (identified by the red dotted line). This clearly establishes the crucial role played by the finite transverse extent of the beam.
