# Supplementary material for: Magnetic turbulence in a table-top laser-plasma relevant to astrophysical scenarios
Source: Nat Commun. 2017 Jun 30;8:15970. doi: 10.1038/ncomms15970 (PMC5497062; doi:10.1038/ncomms15970)
Supplement: Peer Review File [file ncomms15970-s4.pdf]

Editorial Note:

This manuscript has been previously reviewed at another journal that is not operating a transparent peer review scheme. This document only contains reviewer comments and rebuttal letters for versions considered at Nature Communications.
